# Study protocol of the ALMA-CKD trial; an electronic triggering decision-support system to improve the detection, recognition, and management of patients with chronic kidney disease in primary care

**DOI:** 10.1186/s12882-024-03852-z

**Published:** 2024-11-13

**Authors:** Jacob Andersson-Emad, Arvid Thunholm, Stephen Nash, Marie Evans, Sara Lind af Hageby, Johan Ärnlöv, Marie Hilderman, Martin Forseth, Arvid Sjölander, Stefan H. Jacobson, Juan Jesus Carrero

**Affiliations:** 1 Department of Neurobiology, Care Sciences and Society, Karolinska Institutet, Stockholm, Sweden; 2 Blackwell Medtech, Stockholm, Sweden; 3 Department of Medical Epidemiology and Biostatistics, Karolinska Institutet, Stockholm, Sweden; 4 Department of Clinical Science, Intervention and Technology, Karolinska Institutet, Stockholm, Sweden; 5 Department of Nephrology, Karolinska University hospital, Stockholm, Sweden; 6 Stockholm läns sjukvårdsområde, Region Stockholm, Stockholm, Sweden; 7 Division of Nephrology, Department of Clinical Sciences, Karolinska Institutet Danderyd Hospital, Stockholm, Sweden

**Keywords:** Chronic kidney disease, Clinical decision support systems, Practice facilitators, Screening, monitoring, awareness, management

## Abstract

**Background:**

Chronic kidney disease (CKD) is a global health problem affected by under-recognition and under-treatment in primary care settings. Electronic clinical decision support (CDS) triggering systems have the potential to improve detection and management of people with CKD by assisting clinicians in adhering to guideline recommendations. We aimed to test whether an electronic CDS triggering system would improve the detection, recognition, and management of patients with CKD in primary care.

**Method/Design:**

This is a pragmatic cluster-randomized controlled trial where 66 primary healthcare centers from the Stockholm Region, Sweden were randomized 1:1 to receive either a new expanded CDS-triggering system offering kidney-specific advice or to continue with their current CDS-triggering system. The expanded CDS system reminds and provides practical facilitators of the processes of CKD screening, recognition with a diagnosis, management and referral to specialist care. The trial duration is 24 months and it is embedded into the Stockholm CREAtinine measurements (SCREAM) project, a repository of healthcare data from the region, which minimizes disturbances with healthcare praxis due to the trial and makes it fully pragmatic. The primary outcomes are the number of eligible patients screened for creatinine and albuminuria once annually and the re-testing of these labs within 6 months in patients with abnormal eGFR or albuminuria. Secondary outcomes are the proportions of issued clinical diagnoses among those fulfilling criteria, proportions of patients with significant albuminuria receiving prescribed nephroprotective medications, proportions of accepted referrals to nephrologist care among those fulfilling criteria and proportion of referrals for ultrasound of the kidneys.

**Discussion:**

Prior pragmatic trials of CDS-systems in CKD has shown an improvement in quality indicators primarily in patients already diagnosed with CKD. This study expands this evidence by focusing on the process of screening, identification, monitoring and diagnostic work-up.

**Conclusion:**

This pragmatic trial will assess the value of CDS for improved adherence to CKD guidelines in primary care. Clinicaltrials.gov registration: NCT06386172, submitted 2024-04-23.

**Supplementary Information:**

The online version contains supplementary material available at 10.1186/s12882-024-03852-z.

## Introduction

About 10–15% of adults are currently estimated to suffer from CKD [1, 2], a condition characterized by reduced kidney function or signs of kidney damage that are sustained over time. Patients with CKD have increased risks of medication errors [3], comorbid complications, all-cause mortality, cardiovascular mortality and morbidity, as well as kidney failure requiring dialysis or transplantation [4].

Many patients with CKD are at low risk of progression to kidney failure and are ideally managed in primary care settings [5]. This is the current model of decentralized CKD care in most health systems including in Sweden. Clinical guidelines [6–8] provide recommendations regarding the screening, identification and management of patients with CKD in primary care, as well as criteria for referral to nephrologist care. The goal of these recommendations is to improve detection and recognition and to reduce the risk of adverse consequences of kidney failure and cardiovascular disease.

Despite the presence of clear guidelines, reports suggest that globally, most adults with CKD remain undetected, undiagnosed, improperly risk stratified, and undertreated [9–11]. The region of Stockholm is not an exception: only 9% of people with laboratory-detected CKD carry a clinical diagnosis or have ever visited a nephrologist [12]. In an evaluation of the processes of CKD care in about 227.000 people with a first-ever identified eGFR < 60 ml/min/1.73 m [2] while attending consultation in primary care [13], we reported that within the following 18 months, only 28% of patients were screened for albuminuria. The rates of testing were higher in patients with diabetes, where guidelines put more emphasis, but still about 50% of patients with diabetes failed to have albuminuria tested. In patients meeting clear criteria for referral to nephrologist care, only 8% of women and 13% of men visited a nephrologist in the following 18 months; of patients with a clear indication for RAS inhibitors, only 46% of women and 56% of men were receiving them.

Barriers that hinder effective CKD identification and care in primary care settings include lack of awareness and/or understanding of guidelines for risk stratification and management of CKD, confusion regarding appropriate referral criteria and timing, lack of confidence in managing CKD and limited communication channels with specialists in nephrology [14]. Additionally, general practitioners may have limited time to manage a complex visit agenda [15]. Programs that facilitate the processes of CKD identification and management and enhanced cooperation between general and specialist care have thus the potential to improve these identified health care gaps.

Widespread use of electronic health records offers new opportunities to identify and address such gaps by implementation of electronic clinical decision support (CDS) systems. CDS systems are designed to aid clinical decision-making during the process of care. When well-designed and effectively used, they can be powerful tools to improve the quality of patient care and prevent errors and omissions [16]. CDS triggers, that is, CDS systems that automatically activate upon a “trigger” (e.g. when the patient journal is opened or a laboratory test is ordered), have advantages compared with CDS systems that require active engagement of the clinician (i.e. having to access a specific website or platform to consult advice). CDS triggers can enable actions and promote proactive preventive care, such as opportunistic disease screening; they can also serve as reminders to staff and clinicians to take action to facilitate care delivery to a patient, alerting them while they’re working in the electronic healthcare records, and linking them to the appropriate place to take the preferred action [17, 18]. 

The region of Stockholm, Sweden, with a population of 2.45 million citizens in 2023 provides universal healthcare with a single unified health system managed by Region Stockholm. Stockholms läns sjukvårdsområde (SLSO) is a section within Region Stockholm responsible for primary care, psychiatry, and medical education training/habilitation. SLSO manages the 66 public non-subsidized primary care centers of the region, providing health services to about 800 000 Stockholm citizens ascribed to them primarily because of geographical proximity, or by an active choice made by the patient. Since November 2022, a platform for CDS triggers called ALMA (Automated Learnable Medical Assistant) has been implemented in these centers and integrated in the electronic healthcare environment. ALMA activates its CDS algorithms when an electronic patient record (i.e. Takecare© in the region of Stockholm) is opened during a healthcare visit, and it can detect issued diagnoses, laboratory test results, prescribed medications and read text. An alert activates when triggering conditions are met, resulting in a pop-up window appearing in the screen with a reminder providing advice and considerations for patient management, in many cases offering to automate processes, like issuing a clinical diagnosis, ordering laboratory tests or referring patient to further investigations [19]. ALMA is a platform that provides advice/guidance to physicians pertinent to multiple diseases and conditions, periodically introducing new advice or modifying existing ones if new clinical guidelines are published.

## Purpose

Few CDS applications have been specifically designed to improve CKD management in primary care and/or to facilitate interactions with nephrology-specialist units. Using an already-established CDS platform, we employed a CDS triggering system of our own design to aid primary care physicians in the process of identification, recognition and management of CKD. We designed a pragmatic cluster-randomized clinical trial to test whether a CDS trigger system will improve the quality of care of people with CKD.

## Materials and methods

### Development of the ALMA-CKD CDS triggering system

**Fig. 1:**
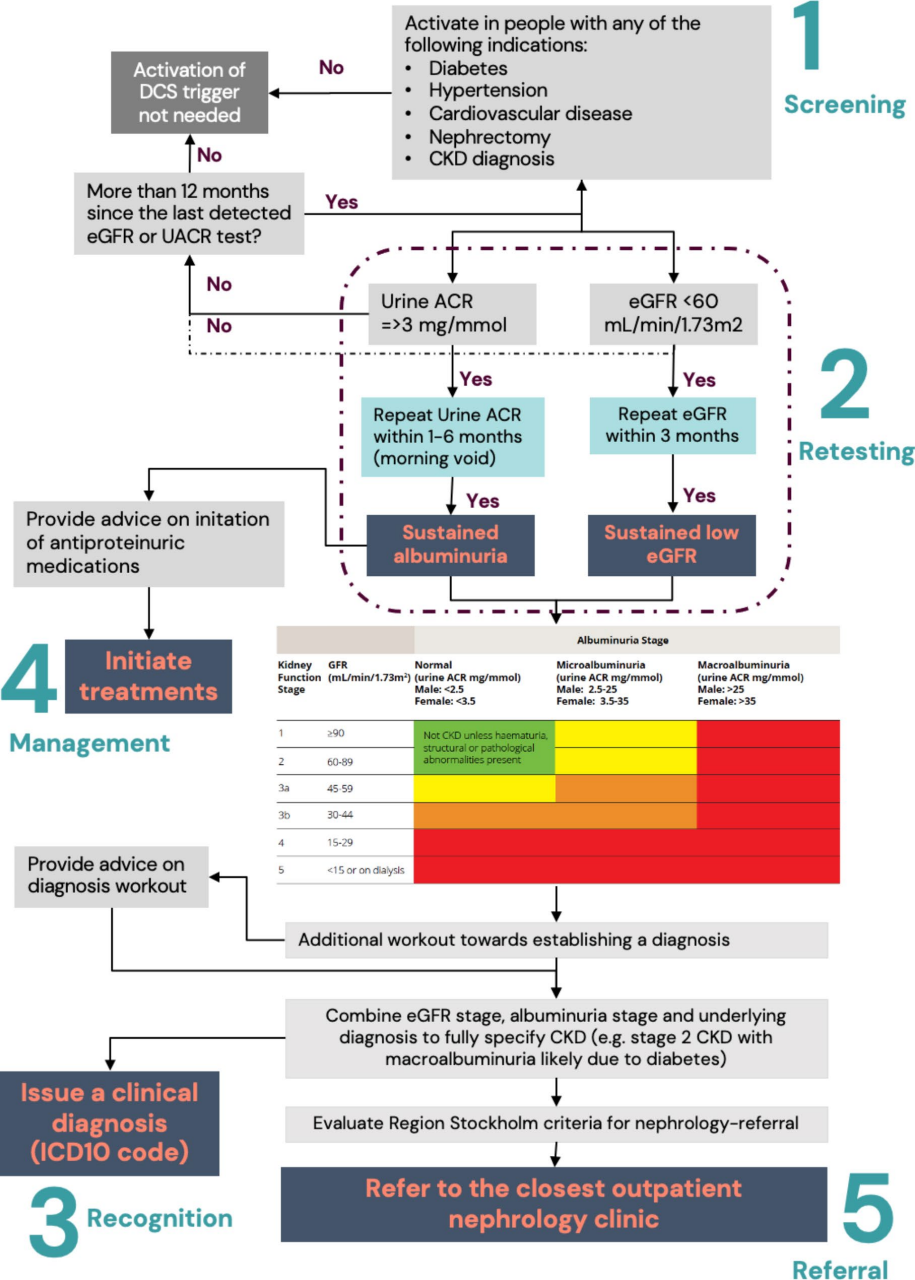
Schematic representation of the five main steps considered for the identification and management of CKD in primary care

We translated the regional [7] and national [6] guidelines for CKD identification and management in primary care into ALMA-triggers and facilitators. This resulted in a battery of CDS triggers grouped into 5 different processes of CKD care (Fig. 1). Selected key examples of how these triggers operate are described in Table 1.

**Table 1:** Selected examples of CDS triggers and advice/practice facilitators offered to the physician

	Example of trigger	Pop-up alert	Information/advice text	Practice facilitator/action
**Step 1: Annual screening for albuminuria and creatinine in populations at risk of CKD**.				
	A female patient has a clinical diagnosis of hypertension in her medical records. The last time that a serum creatinine or a urine albumin test appears in her medical records is more than 12 months ago.	Need to monitor kidney function	The patient has or it at risk of developing CKD. The kidney function has not been monitored during the last 12 months. Do you want to order testing with creatinine and uACR?	•Click here to automatically order a test for creatinine and uACR
**Step 2: Re-testing of abnormal kidney function tests**				
	The laboratory test results ordered during Step 1 are ready and as follows: UACR < = 3 mg/mmol and eGFR 42 ml/min/1.73 m^2^.	Possible risk of CKD	The last eGFR test indicates some degree of kidney damage. According to current guidelines you should control this with a new test within 1–6 months	•Click here to automatically add a new test for creatinine and UACR within approximately 3 months
**Step 3: Considerations towards establishing a clinical diagnosis.**				
	The laboratory test results ordered during Step 2 are ready continue to show a low eGFR of 44 ml/min/1.73 m^2^.	Your patient has CKD stage 3	According to current guidelines your patient has chronic kidney disease stage 3 with a sustained eGFR < 60 ml/min/1.72 m^2^. Do you want to issue a diagnosis? You should also consider a basic investigation of kidney disease according to viss.nu if it has not been performed	•Click here to automatically issue a clinical diagnosis of CKD stage 3 •Click here to automatically order the laboratory test package for kidney disease investigation •Click here to automatically order a kidney ultrasound with prespecified referral comments regarding investigation for CKD
**Step 4: Use of CKD-modifying agents**				
	A patient has a recorded dipstick albuminuria or UACR test indicating > 300 mg/ mmol less than 12 months ago, has a recorded prescription of ACEi/ARB less than 12 months ago, but has no recorded prescription of SGLT-2i	Intensify treatment for albuminuria	Your patient has macroalbuminuria. According to current guidelines, you should consider prescribing an SGLT2-i in addition to treatment with ACEi/ARB. Do you want to prescribe an SGLT2-i?	•Click here to automatically issue a SGLT2-I prescription
**Step 5: Considerations for referral to nephrology-specialist care.**				
	A 50-year-old male has two consecutive recorded tests of macroalbuminuria a dipstick positive for hemoglobin	Consider referral to nephrology care	Your patient has macroalbuminuria, microhematuria and is < 55 years old. According to current guidelines you may consider referring this patient to a nephrology unit for his/her CKD. You should also consider performing a basic laboratory test investigation of CKD if this has not been performed before. If you patient has diabetes and HbA1c is not optimized, consider intensifying the treatment.	•Click here to automatically order the laboratory test package for kidney disease investigation •Click here to automatically order a kidney ultrasound with prespecified referral comments regarding investigation for kidney disease •Click here to automatically create a referral letter to the nephrology unit for you to approve

The CDS triggers were developed by a multidisciplinary group of nephrologists, primary care physicians and software developers. Software engineers at ALMA developed the CDS triggering algorithms that were internally tested in an artificial environment. As a next step, the CDS triggers were tested in a pilot phase at Gustavsberg academic primary healthcare center. Physicians at that center experienced the CDS triggers during a 2-week period, provided feedback on its functionality and gave suggestions for improvement of it. We then determined that the CDS-triggering system was ready for evaluation in a clinical trial.

### Trial design

This is a pragmatic cluster-randomized, parallel, two-arm controlled clinical trial (ClinicalTrials.gov ID NCT06386172) involving all public non-subsidized primary care centers in the region of Stockholm (*n* = 66) with the goal to compare the usefulness of ALMA-CKD triggers versus the routinely issued CKD advice currently provided in the ALMA platform. Retrospective registration of the trial protocol was first posted on clinicaltrials.gov on 2024-04-26. The trial sponsor is Karolinska Institutet.

### Trial arms

The 66 primary care centers that used the ALMA platform were randomized in proportion 1:1 to:

- Control arm: Be exposed to the advice/support already-available in the ALMA platform regarding management of kidney disease that consists of: a)Recommendation to test for albuminuria once annually in patients with diabetes; b)Recommendation to start ACE-inhibitors in patients with diabetes and albuminuria; c)Recommendation to establish a diagnosis of diabetic nephropathy in patients with diabetes and eGFR < 60 ml/min/1.73 m [2] or albuminuria; and d) alert if there is a recorded decrease of eGFR of more than 20% in the last 18 months.
- Intervention arm: In addition to the advice already provided by the ALMA platform, be exposed to additional advice/support related to the management of CKD (ALMA-CKD CDS triggering system).

Clinicians working at the healthcare centers will be blinded as to which arm the center has been allocated. The research team will be blinded, other than members who work for Blackwell Medtech and that by necessity must implement the intervention. There are no plans to unblind center staff or the research team.

The randomization procedure is described below.

### Study endpoints

Study outcomes are defined at the level of individual patients.

The primary outcomes are creatinine and albuminuria screening within 12 months among those with an indication. For clarity, for each patient visiting one of the participating centers, and who meets the indication for annual kidney function screening, we will evaluate and compare whether they received a screening test for these biomarkers. We will evaluate the testing of each filtration marker separately. Patients eligible for screening are, according to current guidelines, those with a diagnosis of CKD, hypertension, diabetes, cardiovascular disease or nephrectomy.

The secondary outcomes refer to subsequent processes of CKD care as follows: For each patient visiting one of the participating centers, who meets the indication for re-testing of creatinine or albuminuria, we will measure whether a re-test was performed within six months of their original test date. An indication for re-testing creatinine is having eGFR < 60 ml/min/1.73 m [2] in the original test, and an indication for re-testing albuminuria is having a dipstick test denoting KDIGO A2 + or a urinary albumin to creatinine (uACR) test > 3 mg/mmol in the original test. This analysis will exclude those participants carrying already a clinical diagnosis of CKD.

For each patient visiting one of the participating centers, who continue to have eGFR < 60 ml/min/1.73 m [2] and/or dipstick A2 + or > 3 mg/mmol at re-testing, we will measure the proportion of issued clinical diagnoses of CKD (by ICD-10th system) within 6 months. This analysis will exclude those participants carrying already a clinical diagnosis of CKD.

For each patient visiting one of the participating centers, who continue to have dipstick A2 + or > 3 mg/mmol at re-testing, we will measure the proportion of patients who start CKD-modifying agents (ACE/ARBs, SGLT2-i, etc.) or, if a CKD-modifying agent was already being dispensed, whether a second line of therapy was started, both within 6 months.

For each patients fulfilling criteria for referral to nephrologist care, we will record the proportion of referrals for ultrasound of the kidneys and the proportion of accepted referrals to nephrologist specialist care within the intervention period. Criteria for referral to nephrologist care are those recommended by Swedish guidelines [6]. This analysis will exclude those participants with already recorded visits to the nephrologist in the 2 years prior to trial start.

### Analysis population and trial duration

As this is a pragmatic trial, all analysis will be performed on an intention to treat population, consisting of all eligible patients, analyzed according to the randomized allocation of the healthcare center. Each individual will only be included in each analysis once; that is, they will be included into the analysis population for each endpoint on the first time they visit a participating center during the recruitment phase and meet the eligibility criteria. If they do not meet the criteria on the first visit, but do on a subsequent visit, they will be included on that second visit, but the same individual will never be included twice in the same analysis set.

All patients who visit a participating healthcare center at some point during the 12 months following randomization will be considered for inclusion, and all will be followed up for a further 12 months. Hence the trial will run for a total of 24 months.

### Activities to enhance use of algorithms

Two initiatives will be implemented to increase awareness of the ALMA-CKD triggers amongst the general practitioners at the centers allocated to the intervention arm. Within 3 months from trial start, an informative video on the new ALMA-CKD features was sent to all attending clinicians and posted in the internal website of the SLSO centers (but only visible by the people employed in the centers randomized to intervention). Within 6 months from trial start, 2 more specific educational videos on how to benefit from ALMA-CKD will be posted in the educational section of the SLSO internal website (again, only visible by the people employed in the centers randomized to intervention) and sent to the managers of respective center for distribution.

### Pragmatic trial with no active data collection

This is a pragmatic trial, and as such we have designed it to minimize interactions between patients and researchers, so as to avoid interfering with routine of care. Patients will receive care according to the decisions of their clinician in their choice of healthcare center and will not be asked to make additional visits or provide additional samples for the purposes of this study.

A novelty of this trial is its embedment in the well-established Stockholm CREAtinine Measurements (SCREAM) data repository [20]). By this feature, the study design resembles a registry-randomized RCT, which is a modern and effective way to run pragmatic trials, benefiting from the excellent information collected in Swedish databases, and minimizing trial costs as there is no need to call participants for programmed visits, checkups or samplings. As such we will not have a Data Monitoring Committee.

SCREAM is a complex linkage of administrative and clinical healthcare databases from Region Stockholm with continuous updates since 2006 [20]. For this trial, the following SCREAM databases needed are:

1. *The administrative health data registry of Region Stockholm* (Vård Analys Databasen, VAL; the Region Stockholm healthcare data warehouse). VAL contains information on all consultations in primary and specialist outpatient care, as well as hospitalizations. Patients visiting the cluster center will be identified through center codes linked to each encounter. VAL also contains issued diagnoses and procedures, which will help us to evaluate eligibility for screening as well as occurrence of new diagnoses of CKD during the trial. VAL also allows identifying accepted referrals to nephrologist care. Finally, other necessary information from VAL includes demographic information (age and sex), migration to/from Stockholm (which allows for censoring participants when their residency changes) and ascribed primary care center (to explore the proportion of patients attending their assigned primary care center or another one).
2. *The repository of laboratory data*. Three laboratory companies (Unilabs, Synlabs and Karolinska) perform the vast majority (> 95%) of clinical chemistry laboratory tests of the region, including the totality of SLSO centers included in our trial. From this repository, we will extract information on all performed analyses of plasma/serum creatinine, cystatin C and albuminuria (dipstick and urinary albumin to creatinine ratio). For each measurement we will have information on the date and time of measurement, units, method used, reference interval, and the department/unit that requested the test. In this way, we can identify with precision which tests were ordered from the centers in our trial.
3. Linkage with two registries coordinated by the Swedish National Board of Health and Welfare: (a) *The Prescribed Drug Registry* [21] containing all pharmacy dispensations of prescribed drugs. This will allow for the evaluation of use or initiation of recommended CKD-modifying agents; and (b) *The Population Registry* [21] with information on vital status and, when applicable, reported cause of death as a censoring event.
4. Via linkage with TakeCare intelligence warehouse, which administers the electronic healthcare records system of the region, we will obtain information on clinical parameters such as height, body weight, blood pressure or smoking habits of patients seeking care in the centres involved in our trial. This will not be necessary to analyse the outcomes of our trial, but it will be used in the next phase where we will explore the impact of CKD recognition and management on health events.

### Sample size

To estimate the required sample size, we evaluated the processes of CKD care in the 66 primary healthcare centers entering this trial during 2019. This information was available to us from the SCREAM repository. We chose the year prior to the COVID-19 pandemic to have more representative estimations of health care use not affected by lock downs and policies during the pandemic. We believe this information is rather unique and provide it as a supplementary information for use by other researchers in their power considerations for future trials (Supplementary table).

During 2019, 662 995 unique individuals visited the primary healthcare centers included in the trial at least once. The size of the centers varied, as well as the number of patients with an indication for CKD screening in them. In addition, creatinine and albuminuria testing and re-testing rates were different across centers. Some primary care centers were in residential communities/neighborhoods of generally older age, whereas other centers tended to receive patients of younger age, hence with less indications for CKD screening. Of all people visiting the health care centers, 92 011 were eligible for screening/testing of kidney function based on their comorbidity profile: 66% took a creatinine test, and 34% took an albuminuria test. Of people with first detected eGFR < 60 ml/min/1.73 m [2] or albuminuria > 30 mg/L, 59% and 28% were retested for creatinine or albuminuria, respectively, within 3–6 months.

Based on the testing and re-testing rates of participating centers in 2019, the study has 85% power to detect an absolute improvement in our primary study outcome of 13% points with an alpha of 5%, and similar power to detect differences in the first three secondary outcomes. The effect size was estimated by assuming that the intervention would increase the proportion being tested by 20–30% (relative increase). For the primary outcome we used a conservative estimate of 20%, hence from 0.66 to 0.79. We assumed a recruitment period of 12 months and a total of 33 health center (clusters) per arm with the same number of patients as in 2019. We estimated the intraclass correlation for the outcomes to be 0.1 (as quantified in the UK trial by Major et al. [22]).

### Randomization

Randomization was restricted to ensure (approximate) balance on two factors that differed across the participating centers: the number of people that visited them in 2019 with an indication for CKD screening, and the proportion of these people that were tested for albuminuria. In total, 62 of the centers had < 3000 patients visiting them in 2019 with an indication for CKD screening. Four centers, however, had a much larger eligible population, with 5434, 4712, 3762, and 3286 patients. We first created 10,000 randomizations of the 62 “non-large” centers, with 31 in each arm. We then created all possible permutations of the four large centers such that two were in one arm and two were in the other arm. There are six possible permutations which achieve this (AABB, ABAB, ABBA, BAAB, BABA, BBAA). These two sets (10,000 randomizations of the 62 large centers, and 6 randomizations of the smaller centers) were combined to give us 60,000 potential randomizations and then we mirrored these allocations to give us 120,000 randomizations (that is, reversed the allocation of intervention and standard arms). For each potential allocation we calculated the average proportion (within each randomization arm) of people tested in 2019. This was calculated on a center level, not a population level, so the denominator was 33 in each arm. If the difference in the average proportion between the arms was greater than 3.7%, we discarded the allocation. The figure of 3.7% was chosen because, under different randomly drawn sets of the 10,000 allocations, 3.7% ensured that approximately, but less than 10% of allocations, were discarded. Furthermore, it was felt that 3.7% was small enough such that if there was indeed a between-arm difference of 3.7% we would still be confident that the two arms were similar enough to be confident of drawing unbiased conclusions form the results. Finally, one allocation was randomly selected from the remaining ~ 108,000 allocations.

The code to perform this randomization was given to an independent statistician, outside of the study team, who changed the random seed that selected the 10,000 allocations, and the random seed that selected the final allocation. When the code was run, 10,348 (8.6%) allocations were removed due to a difference between the arms in 2019 test rates.

### Post-randomization change and sample size re-estimation

Four days after performing this randomization, the study team was informed that two of the 66 centers had been merged into one administrative unit (although still being two geographically separate sites). Due to this merge, we will not be able to retrieve outcome data at a lower level than the merged center. As by chance these two centers had been assigned to the same arm, we decided to proceed with the trial under the original randomization. We note here that the two original restrictions still held after the merger of these centers: the eligible population in 2019 of the new merged center was 1,455, which means it ranked 24th (of 65) in size; the difference between the two arms in terms of 2019 test rates became 3.4% (< 3.7%). The trial will then analyze 65 centers (33 in the standard arm and 32 in the intervention). Table 2 gives the originally calculated power, and the power that we now have after the administrative merging of two centers, conservatively assuming 32 clusters per arm.

**Table 2:** Power calculation

Comparison	Original power (33 clusters per arm)	New power (32 clusters per arm)
A. Detect an increase in proportion of people having a creatinine test from 66–79%	86%	**85%**
B. Detect an increase in proportion people having an albuminuria test from 34–48%	84%	**83%**
C. Detect an increase in proportion of people having a re-test of creatinine from 59–73%	86%	**82%**
D. Detect an increase in proportion of people having a re-test of albuminuria from 28–42%	86%	**84%**

### Statistical analysis

All analyses will be performed on an ITT basis, where each center is analyzed according to the arm it was assigned to at randomization. The primary analyses will be performed at the level of the individual patient. We will use logistic regression, with a random effect to account for the clustering by healthcare center and including a binary variable to indicate randomized treatment allocation. We will additionally include the baseline level of testing in the center (proportion in 2019), and a binary variable indicating the size of the center (large or not-large), as both of these were used in the randomization procedure. This analysis will produce a relative measure of effect, namely an odds ratio. We will also present an absolute measure of the intervention effect.

The primary outcome will also be analyzed at the center level, adjusted for the same variables described above: 2019 testing proportion and center size.

The same approach will be used for the secondary outcomes. To test whether repeated clinic visits lead to changed behaviour, we will perform two subgroup analyses. First, by the number of visits a participant makes during the recruitment phase: 1 visit vs. more than 1 visit. Second, by sex: men vs. women. Exploratory analysis will use a time-to-event outcome for each participant, instead of a simple binary variable calculated at 12 months. No other pre-planned subgroup analyses will be performed, nor are any interim analyses planned. As this study uses routinely collected data we do not anticipate missingness and have no plans to impute missing data. A detailed description of the methods to be used will be given in a Statistical Analysis Plan, which will be uploaded to clinicialtrials.gov prior to the time of last data collection and before any analysis has been performed.

### Declarations

#### Ethics approval and consent to participate

We have obtained approval to conduct this trial by the Swedish Ethical Review Authority (EtikPrövningsMyndigheten, EPM, registration number 2023-03537-01). The EPM will be informed of any changes in the study protocol in accordance with applicable requirements. Because the goal of this pragmatic intervention is to improve current clinical practice by ensuring adherence to current recommendations and protect participants from potential risks of CKD underdetection, it was deemed by the Swedish Ethical Review Authority that it was not necessary to obtain informed consent from individual patients potentially accessing those primary care centers during the study. We contacted the head of all 66 primary healthcare centers to inform them of this trial and give them the option of having their center opting out. Patients at sites randomized to standard of care/no extended ALMA will obtain health care through currently available best clinical practice.

1. **Data management and data access**.

Data will be analyzed and stored at the Department of Medical Epidemiology and Biostatistics at Karolinska Institutet. The trial dataset will not be available for public use as it would breach GDPR regulations of patient’s privacy. Data can be available on reasonable request to investigators for proposals that comply with GDPR, national and institutional regulations for data sharing and collaborative research.

2. **Dissemination policy**.

We will present results of the study to Region Stockholm and the participating centers and seek to present and publish results of the intervention on the outcomes described here at national/international conferences and in a peer-reviewed scientific journal. Authorship will be decided on intellectual contribution to the research (and not due to any financial incentives), and we have no plans to use professional writers.

3. **Steering committee**.

Decisions regarding the conduct of the trial, data management and analysis will be considered by the Trial Steering Committee, which consists of Profs. Juan J Carrero, Stefan Jacobsson and Arvid Sjölander, Assoc. Prof Marie Evans, Dr. Jacob Andersson-Emad and Stephen Nash. As all participants will receive standard care under the direction of their regular clinician we do not anticipate adverse events and do not require a Data Monitoring Committee.

4. **Funding declaration**.

The study is financed by grants from the Swedish state under the agreement between the Swedish government and the county councils, the ALF-agreement (FoUI-986028), and by the Swedish Research Council (2023 − 01807). We also acknowledge donations from AstraZeneca and Boehringer Ingelheim to Danderyd University Hospital. The funders have no role in the design and conduct of the study; collection, management, analysis, and interpretation of the data; preparation, review, or approval of the manuscript; and decision to submit the manuscript for publication.

#### Competing interests

Arvid Thunholm is an employee of Blackwell Medtech, the company that developed and manages the ALMA platform. None of the rest of investigators involved in the study have any financial interest or possible benefit from the results of this trial.

5. **Author contributions and consent for publication**.

Study concept: J.J and S.H.J; Writing of the first draft: J.J.C and J.A.E; First round of revisions: S.N; Second round of revisions and final approval of the Ms: Rest of authors. All authors consent to submit this Ms for publication.

## Discussion

Here we present the protocol for a cluster-randomized, two-arm, pragmatic trial which aims to demonstrate that a CDS triggering system can improve essential processes of care for the identification and management of CKD. As such, results have direct implications for the health care of CKD in Region Stockholm, translating evidence into practice and aiming to improve health outcomes for these patients. Evidence from this trial will also have implications for other health systems worldwide that would like to implement similar measures, particularly in health delivery systems that care for patient populations with a high burden of CKD.

Several pragmatic trials have explored similar research questions to ours in other health systems, all of them summarized in Table 3 [22–27]. In general, most of them were performed in primary care settings and demonstrated effectiveness of a CDS system in improving quality indicators of CKD care, primarily a higher awareness or higher proportion of people with a CKD diagnosis. However, most trials did not evaluate or did not observe differences in patient clinical outcomes after these improvement of quality indicators. A potential limitation of some trials is short duration (12 months or less), low sample size and that they were performed prior to the addition of SGLT-2i in the guidelines for CKD. The longest trial (42 months) was done by Major et al. [22] which employed a CDS system with nurse-led assistance, and during that period they failed to observe any difference in the change from baseline of the mean eGFR at a cluster level (at a care center level). Due to limitations of data access in these primary care centers, the authors were not able to evaluate individual changes in eGFR. Given the inherent variability of creatinine-based eGFR and how it may be affected by disease burden (i.e. frailty), we reflect that mean eGFR change at a center level is a rough, probably insensitive outcome that does not preclude many individuals benefiting from the intervention. Also, some of the nephroprotective drugs such as SGLT2 and RAS inhibitors, may lead to short-term drop in eGFR, but a long-term benefit with a slower progression. The second longest trial (36 months) was by Carroll et al. [26]used a CDS system with practice facilitators and reported a slower annual loss of eGFR, but counterintuitively indicators of CKD care (implementation of guideline-recommended treatments, issuing CKD diagnoses, etc.) did not change. Some trials focused on specific subpopulations, such as patients with both CKD and hypertension, and all of them aimed at improving care in populations with manifest CKD. We are not aware of studies that aim at identifying CKD patients (i.e., screening and retesting in people with established risk factors) and our trial will add novel information to this field. While the value and cost/effectiveness of universal or home-based screening for CKD is still debated [28] investigations in our own health system [13] and globally [9–11, 29] show that there is an opportunity for early detection, risk-stratification and monitoring of CKD.

**Table 3:** Summary of trials investigating CDS systems aimed at CKD-management

Year PMID Author	Country	Design	Intervention	Outcomes	Duration	Arms	Main Findings	Identified challenges
2011, Abdel-Kader et al. [23]	USA	Cluster-randomized controlled trial (single center)	Real-time automated electronic medical record alerts vs. usual care in patients with CKD	Nephrology referral, proteinuria assessment, CKD documentation, blood pressure control	10 months	30 GPs randomized. 248 patients in total,	No significant differences in primary outcomes; trends towards improved proteinuria assessment and blood pressure control	Small sample size, single-center study, passive alerts (no triggers)
2018, Carroll et al. [26]	USA	Cluster randomized controlled trial (multicenter)	A CDS system without (control) or with practice facilitation (intervention) in patients with CKD	Annual loss in eGFR. Indicators of CKD care	36 months	10 centers in control arm, 20 centers in intervention arm. 6699 patients in total	Slower annual loss of eGFR, Better HbA1c control. No difference in CKD diagnosis, NSAID avoidance, use of ACE/ARB	Large dropout of control-centers due to changes in the electronic medical record system during the trial. eGFR decline changed, but not its management
2019, Major et al. [22]	UK	Cluster randomized controlled trial (multicenter)	Nurse-led interventions vs. usual care in patients with CKD	Change in the (cluster) mean eGFR of the CKD patients at each cluster. Indicators of CKD care.	42 months	23 centers in each arm. 23,357 patients in total	No difference in eGFR at a cluster level; higher number of CKD diagnoses and improved BP control	Limited data access making individual eGFR estimates impossible,
2020, Peralta et al. [24]	USA	Pragmatic randomized controlled trial (multicenter)	Two CDS systems (with or without pharmacy support) vs. usual care in patients with CKD	12-month change in BP. Indicators of CKD care with focus on medication use	12 months	80 physicians randomized. 524 patients in total.	No change in BP or medication use. Higher number of CKD diagnoses in CDS systems vs. usual care	Short trial duration. Low sample size, low recruitment and low testing rates.
2024, Vazquez et al. [25]	USA	Pragmatic open-label, cluster-randomized trial (multicenter)	CDS system vs. usual care in patients with concurrent CKD, hypertension and diabetes	Hospitalization for any case. Emergency department visits, readmissions, cardiovascular events, dialysis, death	12 months	141 centers randomized, 11,182 patients in total.	No change in hospitalization rates; no change in secondary outcomes	Hard endpoints perhaps unlikely to be affected by improved recognition/management in the short term.
2024, Samal et al. [27]	USA	Randomized controlled trial (multicenter)	CDS vs. usual care in patients with concurrent hypertension and CKD	Change in systolic BP within 180 days. Attainment of BP targets, indicators of CKD/hypertension care	12 months	174 GPs randomized. 2026 patients in total	Significant reduction of systolic blood pressure; Better indicators of care. No change in BP target attainment.	Potential variability in care delivery across practices, limited follow-up duration.

An identified risk is that there may be insufficient resources to handle an increased number of referrals to nephrologist care. In an internal, unpublished evaluation, we estimated however that 56% of the referrals to the outpatient clinic of Danderyd Hospital could have been avoided with appropriate use of our CDS triggers. While we hope that this intervention will result in better-justified referrals, we expect a considerable increase in the referral rate, given that the population screened and re-tested will increase. In order to anticipate this and respond to the increased need for specialist care advice, we have ensured additional person time of the nephrologists involved in the referral centers of the region (Karolinska Huddinge, Danderyd and Rosenlund clinics) that will dedicate one additional afternoon per week if needed to respond to and plan the workflow, ensuring back-communication and provision of feedback/advice to the general practitioners as well as adequate care to the patients referred.

Some prior trials have been hindered by alert fatigue, that is, the clinicians stop reacting to the many pop-up windows and alarm signals. This risk cannot be avoided and will by itself constitute an important outcome of the trial. In a narrative review by Alexiuk et al. [30] propose the embedding of clinical guidelines and automated calculations as a potential solution to “alert fatigue” by reducing the GP-workload so that they see the CDS as a facilitator. We have tried to the best of our technical capabilities to do this, including automatization of the processes of scheduling labs or visits, prescribing treatments or populating the referral letter for them. We will obtain trimestral statistics as to how many visits have resulted in the activation of our CDS trigger and how many physicians have chosen to disconnect themselves from the ALMA platform at each center. We will use these reports as an indirect measure of alert fatigue and explore trends in use of our CDS trigger over the course of the trial. A limitation is, however, that we will not be able to identify if a patient changes from a care center from the intervention arm to a center from the control arm (and vice versa).

Some prior trials have also reported difficulties due to the lack of individualization of recommendations that are actionable by the general practitioner, limited focus on education for clinicians, and limited pretrial design phases to allow close integration into the primary care clinical workflow. We have tried to overcome these identified limitations by: (a) Pre-testing the CDS trigger in a pilot phase to ensure integration and operationalizability, as well as aligning the CDS trigger with current national guidelines; (b) providing actionable steps (click buttons) that facilitate the work for the general practitioner, automating actions and perhaps saving a couple of minutes of administration work per patient; (c) providing educational videos about how to use and benefit from the CDS triggers; and (d) ensuring availability of dedicated nephrologists in the outpatient clinics to review referrals and answer consultations.

The knowledge we can derive from this project will not end up when the trial is closed. As discussed above, previous trials evaluating CDS triggers have mostly demonstrated effectiveness on improving clinician execution ) but have not been able to evaluate the impact of this improved execution on patient outcomes because of limited access to information or short trial duration. By having the trial embedded in the SCREAM project, we will be able to follow the patients after trial completion for future analyses that can evaluate at an individual patient level the impact of adequate identification and recognition of CKD on subsequent health outcomes. These outcomes include among others, their trajectories of kidney function decline (i.e. eGFR decline and albuminuria progression); Their metabolic control (e.g. blood pressure control, glycemic control etc.); Their risk of medication errors, such as prescription of nephrotoxic drugs, or incorrect dose adjustments; Adverse health outcomes, such as progression to chronic dialysis, acute kidney injury, major adverse cardiovascular events, hospitalizations, infections etc.; and the value of directed screening for albuminuria/eGFR in populations at high risk of CKD.

Since our trial essentially evaluates the value of supporting tools to increase the detection and recognition of CKD, and because this problem exists in all societies, we hope that our findings will be generalizable to the medical community. However, whether CDS triggers are the best tool to achieve this in all health systems cannot be derived from this study, as clinical praxis is different across regions and countries. For example, it could be that physicians in other regions or countries are less likely to use CDS trigger systems compared to Region Stockholm, or that other health systems do not have an electronic health record’s platform that at present allows the coupling of CDS tools.

## Electronic supplementary material

Below is the link to the electronic supplementary material.

Supplementary Material 1

## Data Availability

No datasets were generated or analysed during the current study.
