# Supplementary material for: Study protocol of the ALMA-CKD trial; an electronic triggering decision-support system to improve the detection, recognition, and management of patients with chronic kidney disease in primary care
Source: BMC Nephrol. 2024 Nov 13;25:408. doi: 10.1186/s12882-024-03852-z (PMC11562349; doi:10.1186/s12882-024-03852-z)
Supplement: Supplementary file 1 — Supplementary Material 1 [file 12882_2024_3852_MOESM1_ESM.docx]

|  | **Overall, number of individuals visited at least in the year 2019** | **Elegible for renal testing (Hx of DM, CVD, HTN). The denominator is total number of people with visits at each center in 2019** | | **Tested for creatinine in the same year (denominator is those eligible for testing)** | | | | **eGFR level (denominator is those tested)** | | | | **Repeated creatinine testing 1-6 months after first creatinine (denominator is those with <60 eGFR)** | | | | **Tested for albumunuria in the same year (denominator is those eligible for testing)** | | | | **A category (denominator is those tested)** | | | | | **Repeated testing 1-6 months after first Albuminuria (denominator is those with A2-3)** | | | |
| --- | --- | --- | --- | --- | --- | --- | --- | --- | --- | --- | --- | --- | --- | --- | --- | --- | --- | --- | --- | --- | --- | --- | --- | --- | --- | --- | --- | --- |
|  |  |  |  | **Yes** | | **No** | | **<60** | | **>=60** | | **Yes** | | **No** | | **Yes** | | **No** | | **A2-A3** | | | **A1** | | **Yes** | | **No** | |
|  | **N** | **n** | **%** | **n** | **%** | **n** | **%** | **n** | **%** | **n** | **%** | **n** | **%** | **n** | **%** | **n** | **%** | **n** | **%** | **n** | **%** | **n** | | **%** | **n** | **%** | **n** | **%** |
| **Overall** | **662,955** | **2,011** | **14** | **60 536** | **66** | **31475** | **34** | **14 773** | **24** | **45 763** | **76** | **8 674** | **59** | **6 099** | **41** | **31 334** | **34** | **60 677** | **66** | **8 877** | **28** | **22 457** | | **72** | **2 462** | **28** | **6 415** | **72** |
| **By center** |  |  |  |  |  |  |  |  |  |  |  |  |  |  |  |  |  |  |  |  |  |  | |  |  |  |  |  |
| 1 | 4 849 | 542 | 11 | 370 | 68 | 172 | 32 | 71 | 19 | 299 | 81 | 27 | 38 | 44 | 62 | 196 | 36 | 346 | 64 | 57 | 29 | 139 | | 71 | 13 | 23 | 44 | 77 |
| 2 | 6 413 | 1 183 | 18 | 627 | 53 | 556 | 47 | 88 | 14 | 539 | 86 | 34 | 39 | 54 | 61 | 202 | 17 | 981 | 83 | 94 | 47 | 108 | | 53 | 25 | 27 | 69 | 73 |
| 3 | 17 412 | 2 526 | 15 | 1 765 | 70 | 761 | 30 | 422 | 24 | 1 343 | 76 | 167 | 40 | 255 | 60 | 757 | 30 | 1 769 | 70 | 208 | 27 | 549 | | 73 | 60 | 29 | 148 | 71 |
| 4 | 14 142 | 2 254 | 16 | 1 511 | 67 | 743 | 33 | 352 | 23 | 1 159 | 77 | 122 | 35 | 230 | 65 | 695 | 31 | 1 559 | 69 | 175 | 25 | 520 | | 75 | 57 | 33 | 118 | 67 |
| 5 | 9 568 | 1 284 | 13 | 932 | 73 | 352 | 27 | 148 | 16 | 784 | 84 | 58 | 39 | 90 | 61 | 486 | 38 | 798 | 62 | 102 | 21 | 384 | | 79 | 22 | 22 | 80 | 78 |
| 6 | 5 571 | 805 | 14 | 502 | 62 | 303 | 38 | 132 | 26 | 370 | 74 | 67 | 51 | 65 | 49 | 375 | 47 | 430 | 53 | 103 | 27 | 272 | | 73 | 38 | 37 | 65 | 63 |
| 7 | 5 257 | 658 | 13 | 470 | 71 | 188 | 29 | 148 | 31 | 322 | 69 | 72 | 49 | 76 | 51 | 228 | 35 | 430 | 65 | 67 | 29 | 161 | | 71 | 14 | 21 | 53 | 79 |
| 8 | 9 968 | 1 556 | 16 | 1 034 | 66 | 522 | 34 | 354 | 34 | 680 | 66 | 173 | 49 | 181 | 51 | 484 | 31 | 1 072 | 69 | 142 | 29 | 342 | | 71 | 23 | 16 | 119 | 84 |
| 9 | 13 847 | 1 879 | 14 | 1 319 | 70 | 560 | 30 | 345 | 26 | 974 | 74 | 128 | 37 | 217 | 63 | 622 | 33 | 1 257 | 67 | 152 | 24 | 470 | | 76 | 47 | 31 | 105 | 69 |
| 10 | 4 350 | 777 | 18 | 515 | 66 | 262 | 34 | 79 | 15 | 436 | 85 | 26 | 33 | 53 | 67 | 262 | 34 | 515 | 66 | 56 | 21 | 206 | | 79 | 20 | 36 | 36 | 64 |
| 11 | 23 089 | 1 976 | 8,60 | 1 329 | 67 | 647 | 33 | 270 | 20 | 1 059 | 80 | 102 | 38 | 168 | 62 | 706 | 36 | 1 270 | 64 | 177 | 25 | 529 | | 75 | 52 | 29 | 125 | 71 |
| 12 | 8 236 | 1 124 | 14 | 886 | 79 | 238 | 21 | 310 | 35 | 576 | 65 | 116 | 37 | 194 | 63 | 386 | 34 | 738 | 66 | 129 | 33 | 257 | | 67 | 41 | 32 | 88 | 68 |
| 13 | 3 102 | 632 | 20 | 415 | 66 | 217 | 34 | 119 | 29 | 296 | 71 | 47 | 39 | 72 | 61 | 372 | 59 | 260 | 41 | 109 | 29 | 263 | | 71 | 46 | 42 | 63 | 58 |
| 14 | 4 246 | 726 | 17 | 544 | 75 | 182 | 25 | 195 | 36 | 349 | 64 | 78 | 40 | 117 | 60 | 293 | 40 | 433 | 60 | 102 | 35 | 191 | | 65 | 35 | 34 | 67 | 66 |
| 15 | 43 946 | 4 712 | 11 | 3 241 | 69 | 1 471 | 31 | 885 | 27 | 2 356 | 73 | 343 | 39 | 542 | 61 | 1 500 | 32 | 3 212 | 68 | 445 | 30 | 1 055 | | 70 | 124 | 28 | 321 | 72 |
| 16 | 5 242 | 702 | 13 | 527 | 75 | 175 | 25 | 142 | 27 | 385 | 73 | 51 | 36 | 91 | 64 | 298 | 42 | 404 | 58 | 93 | 31 | 205 | | 69 | 13 | 14 | 80 | 86 |
| 17 | 4 194 | 302 | 7,20 | 134 | 44 | 168 | 56 | 21 | 16 | 113 | 84 | 12 | 57 | 9 | 43 | 81 | 27 | 221 | 73 | 26 | 32 | 55 | | 68 | 9 | 35 | 17 | 65 |
| 18 | 3 310 | 613 | 19 | 429 | 70 | 184 | 30 | 110 | 26 | 319 | 74 | 49 | 45 | 61 | 55 | 238 | 39 | 375 | 61 | 76 | 32 | 162 | | 68 | 24 | 32 | 52 | 68 |
| 19 | 4 309 | 782 | 18 | 489 | 63 | 293 | 37 | 68 | 14 | 421 | 86 | 26 | 38 | 42 | 62 | 303 | 39 | 479 | 61 | 89 | 29 | 214 | | 71 | 20 | 22 | 69 | 78 |
| 20 | 9 369 | 1 580 | 17 | 993 | 63 | 587 | 37 | 175 | 18 | 818 | 82 | 63 | 36 | 112 | 64 | 472 | 30 | 1 108 | 70 | 123 | 26 | 349 | | 74 | 42 | 34 | 81 | 66 |
| 21 | 5 500 | 834 | 15 | 588 | 71 | 246 | 29 | 172 | 29 | 416 | 71 | 70 | 41 | 102 | 59 | 323 | 39 | 511 | 61 | 94 | 29 | 229 | | 71 | 24 | 26 | 70 | 74 |
| 22 | 8 746 | 1 620 | 19 | 976 | 60 | 644 | 40 | 325 | 33 | 651 | 67 | 142 | 44 | 183 | 56 | 368 | 23 | 1 252 | 77 | 104 | 28 | 264 | | 72 | 22 | 21 | 82 | 79 |
| 23 | 7 063 | 849 | 12 | 481 | 57 | 368 | 43 | 71 | 15 | 410 | 85 | 34 | 48 | 37 | 52 | 230 | 27 | 619 | 73 | 51 | 22 | 179 | | 78 | 11 | 22 | 40 | 78 |
| 24 | 19 624 | 2 848 | 15 | 1 992 | 70 | 856 | 30 | 581 | 29 | 1 411 | 71 | 225 | 39 | 356 | 61 | 1 210 | 42 | 1 638 | 58 | 318 | 26 | 892 | | 74 | 95 | 30 | 223 | 70 |
| 25 | 2 805 | 465 | 17 | 354 | 76 | 111 | 24 | 105 | 30 | 249 | 70 | 43 | 41 | 62 | 59 | 257 | 55 | 208 | 45 | 101 | 39 | 156 | | 61 | 38 | 38 | 63 | 62 |
| 26 | 8 709 | 1 649 | 19 | 1 085 | 66 | 564 | 34 | 279 | 26 | 806 | 74 | 119 | 43 | 160 | 57 | 471 | 29 | 1 178 | 71 | 159 | 34 | 312 | | 66 | 50 | 31 | 109 | 69 |
| 27 | 12 191 | 2 371 | 19 | 1 247 | 53 | 1124 | 47 | 211 | 17 | 1 036 | 83 | 76 | 36 | 135 | 64 | 566 | 24 | 1 805 | 76 | 176 | 31 | 390 | | 69 | 46 | 26 | 130 | 74 |
| 28 | 11 818 | 1 936 | 16 | 1 343 | 69 | 593 | 31 | 305 | 23 | 1 038 | 77 | 130 | 43 | 175 | 57 | 732 | 38 | 1 204 | 62 | 211 | 29 | 521 | | 71 | 73 | 35 | 138 | 65 |
| 29 | 6 099 | 688 | 11 | 446 | 65 | 242 | 35 | 94 | 21 | 352 | 79 | 43 | 46 | 51 | 54 | 146 | 21 | 542 | 79 | 25 | 17 | 121 | | 83 | 8 | 32 | 17 | 68 |
| 30 | 9 150 | 1 450 | 16 | 969 | 67 | 481 | 33 | 303 | 31 | 666 | 69 | 131 | 43 | 172 | 57 | 484 | 33 | 966 | 67 | 141 | 29 | 343 | | 71 | 38 | 27 | 103 | 73 |
| 31 | 28 494 | 2 672 | 9,40 | 1 602 | 60 | 1070 | 40 | 338 | 21 | 1 264 | 79 | 113 | 33 | 225 | 67 | 861 | 32 | 1 811 | 68 | 205 | 24 | 656 | | 76 | 69 | 34 | 136 | 66 |
| 32 | 4 560 | 780 | 17 | 543 | 70 | 237 | 30 | 88 | 16 | 455 | 84 | 45 | 51 | 43 | 49 | 368 | 47 | 412 | 53 | 110 | 30 | 258 | | 70 | 15 | 14 | 95 | 86 |
| 33 | 5 962 | 1 087 | 18 | 752 | 69 | 335 | 31 | 217 | 29 | 535 | 71 | 99 | 46 | 118 | 54 | 449 | 41 | 638 | 59 | 124 | 28 | 325 | | 72 | 30 | 24 | 94 | 76 |
| 34 | 43 514 | 5 434 | 12 | 3 555 | 65 | 1 879 | 35 | 977 | 27 | 2 578 | 73 | 417 | 43 | 560 | 57 | 1 832 | 34 | 3 602 | 66 | 518 | 28 | 1 314 | | 72 | 133 | 26 | 385 | 74 |
| 35 | 4 529 | 570 | 13 | 423 | 74 | 147 | 26 | 101 | 24 | 322 | 76 | 33 | 33 | 68 | 67 | 216 | 38 | 354 | 62 | 71 | 33 | 145 | | 67 | 24 | 34 | 47 | 66 |
| 36 | 6 712 | 1 131 | 17 | 724 | 64 | 407 | 36 | 214 | 30 | 510 | 70 | 101 | 47 | 113 | 53 | 498 | 44 | 633 | 56 | 112 | 22 | 386 | | 78 | 29 | 26 | 83 | 74 |
| 37 | 21 463 | 3 286 | 15 | 1 883 | 57 | 1 403 | 43 | 489 | 26 | 1 394 | 74 | 234 | 48 | 255 | 52 | 1 120 | 34 | 2 166 | 66 | 319 | 28 | 801 | | 72 | 104 | 33 | 215 | 67 |
| 38 | 9 251 | 1 284 | 14 | 828 | 64 | 456 | 36 | 128 | 15 | 700 | 85 | 55 | 43 | 73 | 57 | 525 | 41 | 759 | 59 | 128 | 24 | 397 | | 76 | 43 | 34 | 85 | 66 |
| 39 | 8 978 | 1 576 | 18 | 879 | 56 | 697 | 44 | 268 | 30 | 611 | 70 | 125 | 47 | 143 | 53 | 388 | 25 | 1 188 | 75 | 128 | 33 | 260 | | 67 | 30 | 23 | 98 | 77 |
| 40 | 36 460 | 3 762 | 10 | 2 591 | 69 | 1171 | 31 | 462 | 18 | 2 129 | 82 | 200 | 43 | 262 | 57 | 1 508 | 40 | 2 254 | 60 | 337 | 22 | 1 171 | | 78 | 84 | 25 | 253 | 75 |
| 41 | 5 860 | 923 | 16 | 502 | 54 | 421 | 46 | 103 | 21 | 399 | 79 | 47 | 46 | 56 | 54 | 249 | 27 | 674 | 73 | 72 | 29 | 177 | | 71 | 20 | 28 | 52 | 72 |
| 42 | 7 359 | 1 362 | 19 | 856 | 63 | 506 | 37 | 207 | 24 | 649 | 76 | 82 | 40 | 125 | 60 | 382 | 28 | 980 | 72 | 92 | 24 | 290 | | 76 | 33 | 36 | 59 | 64 |
| 43 | 4 980 | 654 | 13 | 506 | 77 | 148 | 23 | 130 | 26 | 376 | 74 | 48 | 37 | 82 | 63 | 296 | 45 | 358 | 55 | 87 | 29 | 209 | | 71 | 17 | 20 | 70 | 80 |
| 44 | 6 434 | 956 | 15 | 702 | 73 | 254 | 27 | 181 | 26 | 521 | 74 | 71 | 39 | 110 | 61 | 360 | 38 | 596 | 62 | 135 | 38 | 225 | | 62 | 52 | 39 | 83 | 61 |
| 45 | 8 041 | 1 693 | 21 | 1 152 | 68 | 541 | 32 | 221 | 19 | 931 | 81 | 104 | 47 | 117 | 53 | 727 | 43 | 966 | 57 | 248 | 34 | 479 | | 66 | 72 | 29 | 176 | 71 |
| 46 | 5 817 | 706 | 12 | 484 | 69 | 222 | 31 | 87 | 18 | 397 | 82 | 32 | 37 | 55 | 63 | 277 | 39 | 429 | 61 | 79 | 29 | 198 | | 71 | 22 | 28 | 57 | 72 |
| 47 | 6 291 | 952 | 15 | 748 | 79 | 204 | 21 | 172 | 23 | 576 | 77 | 70 | 41 | 102 | 59 | 487 | 51 | 465 | 49 | 144 | 30 | 343 | | 70 | 31 | 22 | 113 | 78 |
| 48 | 4 083 | 678 | 17 | 392 | 58 | 286 | 42 | 59 | 15 | 333 | 85 | 23 | 39 | 36 | 61 | 163 | 24 | 515 | 76 | 41 | 25 | 122 | | 75 | 12 | 29 | 29 | 71 |
| 49 | 4 831 | 677 | 14 | 304 | 45 | 373 | 55 | 72 | 24 | 232 | 76 | 29 | 40 | 43 | 60 | 116 | 17 | 561 | 83 | 36 | 31 | 80 | | 69 | 6 | 17 | 30 | 83 |
| 50 | 3 945 | 574 | 15 | 412 | 72 | 162 | 28 | 135 | 33 | 277 | 67 | 54 | 40 | 81 | 60 | 225 | 39 | 349 | 61 | 74 | 33 | 151 | | 67 | 11 | 15 | 63 | 85 |
| 51 | 13 552 | 1 167 | 8,60 | 771 | 66 | 396 | 34 | 137 | 18 | 634 | 82 | 55 | 40 | 82 | 60 | 448 | 38 | 719 | 62 | 99 | 22 | 349 | | 78 | 38 | 38 | 61 | 62 |
| 52 | 13 069 | 1 072 | 8,20 | 744 | 69 | 328 | 31 | 206 | 28 | 538 | 72 | 81 | 39 | 125 | 61 | 343 | 32 | 729 | 68 | 119 | 35 | 224 | | 65 | 35 | 29 | 84 | 71 |
| 53 | 9 887 | 1 495 | 15 | 1 006 | 67 | 489 | 33 | 204 | 20 | 802 | 80 | 92 | 45 | 112 | 55 | 561 | 38 | 934 | 62 | 140 | 25 | 421 | | 75 | 27 | 19 | 113 | 81 |
| 54 | 3 058 | 319 | 10 | 251 | 79 | 68 | 21 | 77 | 31 | 174 | 69 | 31 | 40 | 46 | 60 | 162 | 51 | 157 | 49 | 40 | 25 | 122 | | 75 | 16 | 40 | 24 | 60 |
| 55 | 7 862 | 814 | 10 | 469 | 58 | 345 | 42 | 102 | 22 | 367 | 78 | 43 | 42 | 59 | 58 | 214 | 26 | 600 | 74 | 58 | 27 | 156 | | 73 | 12 | 21 | 46 | 79 |
| 56 | 15 789 | 2 621 | 17 | 1 776 | 68 | 845 | 32 | 597 | 34 | 1 179 | 66 | 251 | 42 | 346 | 58 | 664 | 25 | 1 957 | 75 | 245 | 37 | 419 | | 63 | 54 | 22 | 191 | 78 |
| 57 | 6 833 | 1 806 | 26 | 1 205 | 67 | 601 | 33 | 285 | 24 | 920 | 76 | 115 | 40 | 170 | 60 | 594 | 33 | 1 212 | 67 | 166 | 28 | 428 | | 72 | 46 | 28 | 120 | 72 |
| 58 | 5 479 | 1 090 | 20 | 723 | 66 | 367 | 34 | 118 | 16 | 605 | 84 | 58 | 49 | 60 | 51 | 532 | 49 | 558 | 51 | 164 | 31 | 368 | | 69 | 29 | 18 | 135 | 82 |
| 59 | 7 446 | 962 | 13 | 696 | 72 | 266 | 28 | 233 | 33 | 463 | 67 | 91 | 39 | 142 | 61 | 438 | 46 | 524 | 54 | 99 | 23 | 339 | | 77 | 42 | 42 | 57 | 58 |
| 60 | 4 914 | 659 | 13 | 452 | 69 | 207 | 31 | 103 | 23 | 349 | 77 | 33 | 32 | 70 | 68 | 137 | 21 | 522 | 79 | 43 | 31 | 94 | | 69 | 14 | 33 | 29 | 67 |
| 61 | 6 108 | 885 | 14 | 547 | 62 | 338 | 38 | 196 | 36 | 351 | 64 | 87 | 44 | 109 | 56 | 197 | 22 | 688 | 78 | 62 | 31 | 135 | | 69 | 18 | 29 | 44 | 71 |
| 62 | 12 930 | 1 895 | 15 | 1 163 | 61 | 732 | 39 | 239 | 21 | 924 | 79 | 76 | 32 | 163 | 68 | 588 | 31 | 1 307 | 69 | 137 | 23 | 451 | | 77 | 41 | 30 | 96 | 70 |
| 63 | 8 288 | 1 247 | 15 | 735 | 59 | 512 | 41 | 142 | 19 | 593 | 81 | 53 | 37 | 89 | 63 | 339 | 27 | 908 | 73 | 82 | 24 | 257 | | 76 | 21 | 26 | 61 | 74 |
| 64 | 19 537 | 2 451 | 13 | 1 766 | 72 | 685 | 28 | 438 | 25 | 1 328 | 75 | 208 | 47 | 230 | 53 | 817 | 33 | 1 634 | 67 | 289 | 35 | 528 | | 65 | 63 | 22 | 226 | 78 |
| 65 | 3 470 | 525 | 15 | 357 | 68 | 168 | 32 | 78 | 22 | 279 | 78 | 37 | 47 | 41 | 53 | 199 | 38 | 326 | 62 | 65 | 33 | 134 | | 67 | 14 | 22 | 51 | 78 |
| 66 | 5 044 | 923 | 18 | 524 | 57 | 399 | 43 | 89 | 17 | 435 | 83 | 32 | 36 | 57 | 64 | 311 | 34 | 612 | 66 | 104 | 33 | 207 | | 67 | 25 | 24 | 79 | 76 |
